# **I**mpact of Including Peritumoral Edema in Radiotherapy Target Volume on Patterns of Failure in Glioblastoma following Temozolomide-based Chemoradiotherapy

**DOI:** 10.1038/srep42148

**Published:** 2017-02-08

**Authors:** Seo Hee Choi, Jun Won Kim, Jee Suk Chang, Jae Ho Cho, Se Hoon Kim, Jong Hee Chang, Chang-Ok Suh

**Affiliations:** 1 Department of Radiation Oncology, Yonsei University College of Medicine, Seoul, Korea; 2 Department of Radiation Oncology, Gangnam Severance Hospital, Yonsei University College of Medicine, Seoul, Korea; 3 Department of Pathology, Yonsei University College of Medicine, Seoul, Korea; 4 Department of Neurosurgery, Yonsei University College of Medicine, Seoul, Korea

## Abstract

We assessed the impact of including peritumoral edema in radiotherapy volumes on recurrence patterns among glioblastoma multiforme (GBM) patients treated with standard chemoradiotherapy (CRT). We analyzed 167 patients with histologically confirmed GBM who received temozolomide (TMZ)-based CRT between May 2006 and November 2012. The study cohort was divided into edema (+) (n = 130) and edema (−) (n = 37) groups, according to whether the entire peritumoral edema was included. At a median follow-up of 20 months (range, 2–99 months), 118 patients (71%) experienced progression/recurrence (infield: 69%; marginal: 26%; outfield: 16%; CSF seeding: 12%). The median overall survival and progression-free survival were 20 months and 15 months, respectively. The marginal failure rate was significantly greater in the edema (−) group (37% vs. 22%, p = 0.050). Among 33 patients who had a favorable prognosis (total resection and MGMT-methylation), the difference in the marginal failure rates was increased (40% vs. 14%, p = 0.138). Meanwhile, treatment of edema did not significantly increase the incidence of pseudoprogression/radiation necrosis (edema (−) 49% vs. (+) 37%, p = 0.253). Inclusion of peritumoral edema in the radiotherapy volume can reduce marginal failures following TMZ-based CRT without increasing pseudoprogression/radiation necrosis.

Currently, standard treatment for glioblastoma (GBM) is surgical resection followed by chemoradiotherapy with temozolomide (TMZ)^1^,^2^. However, the optimal radiation treatment volume is still a matter of debate. Radiotherapy fields and treatment volumes have evolved since the 1970s when whole-brain radiotherapy was considered the standard therapy^3^. Several studies demonstrated that disease progression was noted even within the region receiving the highest radiation dose among patients with a total brain dose of 60 Gy. As a result, a smaller volume than whole-brain has been proposed to reduce toxicity^4^,^5^. Multiple autopsy series and clinical studies demonstrated that tumor progression is predominantly within 2–3 cm of the primary tumor bed^6^,^7^, and a smaller radiotherapy volume limited to the tumor bed does not show any apparent impact on survival or change the failure pattern in the radiotherapy-alone era^6^,^7^,^8^,^9^,^10^. These findings led to the adaptation of 2–3 cm radiation margins by many groups, including the European Organization for Research and Treatment of Cancer (EORTC)^1^,^2^. On the other hand, other groups, including the Radiation Therapy Oncology Group (RTOG) still used 2 cm margins beyond the extent of the peritumoral edema^11^. This was based on post-mortem studies confirming tumor cells within the peritumoral edema^12^, and a margin of 3 cm beyond the edema was proposed as an optimal margin to ensure complete coverage of all tumor cells^13^.

In the era of concurrent chemoradiotherapy with TMZ, many studies have reported that local recurrence is still a major failure pattern in limited-margin radiotherapy. Recently, several studies reported improved local control but higher rates of distant failures in patients with favorable prognostic factors including O^6^-methylguanine-DNA methyltransferase (MGMT) promoter methylation or extensive surgical resection^14^,^15^,^16^,^17^,^18^. However, there is no report so far concerning the relationship between radiotherapy volume and these prognostic factors.

In our institution, most patients were treated with small-field radiotherapy until 2008, as we followed the radiotherapy guidelines of RTOG 98–03^19^. We reported favorable outcomes with our standard chemoradiotherapy^20^. However, with an increasing rate of gross total/subtotal resection and an empirical experience of frequent out-field recurrence without in-field recurrence, we have changed to larger-field radiotherapy including the peritumoral edema.

We assessed the impact of including the peritumoral edema in radiotherapy volumes on recurrence patterns according to extent of surgical resection and MGMT promoter methylation status among GBM patients treated with the standard chemoradiotherapy in a single institution cohort.

## Methods

### Patients

Between May 2006 and November 2012, 179 consecutive patients with histologically confirmed GBM were treated with TMZ-based adjuvant chemoradiotherapy. We selected 167 patients for our analysis, excluding five patients who were initially diagnosed with leptomeningeal seeding and seven patients who received whole-brain radiotherapy for gliomatosis cerebri or extensive disease. MGMT gene promoter methylation status was determined for 145 patients (87%) using a methylation-specific polymerase chain reaction as preciously described^20^ (Table 1). The study protocol conformed to the ethical guidelines of the 1975 Declaration of Helsinki, as revised in 1983, and was approved by Institutional review board (IRB) of Yonsei University Health System. The patient records/information was anonymized and de-identified prior to analysis, and informed consent was not obtained from each participants.

### Treatment

Surgical tumor resection was performed in 150 patients (90%), and 17 patients (10%) underwent stereotactic biopsy. The extent of surgical resection (EOR) in each patient was evaluated using operative findings and postoperative brain magnetic resonance imaging (MRI) reviewed by the radiologist, neurosurgeon, and radiation oncologist. A postoperative MRI was obtained within 48 hours after surgery for 96% (n = 135) patients. EOR was categorized as gross total resection (GTR), subtotal resection (STR), and partial resection (PR). Resection of a gross tumor by 90% or more was defined as STR, and less than 90% defined as PR. GTR and STR were achieved in 47 and 29% of all patients, respectively.

All of the patients received concurrent chemoradiotherapy with TMZ (75 mg/m^2^ of body-surface area per day, 7 days per week from the first to the last day of radiotherapy), followed by six cycles of adjuvant TMZ (150–200 mg/m^2^ for 5 days during each 28-day cycle). Radiotherapy was initiated 10 to 43 days (median, 19 days) after surgery.

Large-field and small-field radiotherapy was used in 108 and 59 patients, respectively. Gross tumor volumes (GTVs) in both fields consisted of the resection cavity and any residual contrast-enhancing tumor on the immediate postoperative MRI. When we delineate GTV, we added 0.5–1 cm margin to compensate irregularity and uncertainty. The clinical target volume (CTV) in the large-field deliberately included peritumoral edema, which is detected on T2-weighted fluid-attenuated inversion recovery (Flair) postoperative MRI images (peritumoral edema + 1–1.5 cm margin). The CTV in the small-field was delineated by adding a 1.5 cm margin to the GTV, regardless of the presence of peritumoral edema. A 3 mm-margin for setup uncertainty was applied to create the planning target volume (PTV) in the 3-dimensional conformal radiotherapy (3D-CRT) plan. No PTV margin was added in the intensity-modulated radiation therapy (IMRT) plan.

One hundred fifty-one patients (90%) were treated with 3D-CRT, and 16 patients (10%) were treated with IMRT. The Pinnacle planning system (Philips Medical Systems, Cleveland, OH) was used for 3D-CRT plans, and the Tomotherapy Hi-Art System (TomoTherapy, Madison, WI) for IMRT plans. The prescribed dose in 3D-CRT was 46 Gy in 23 fractions to the CTV and a boost to the GTV of 14 Gy in seven fractions. In IMRT plans, 60 Gy in 30 fractions was prescribed to GTV and 51 Gy in 30 fractions to CTV using a simultaneous-integrated boost (SIB) technique. The median total radiation dose was 60 Gy (range 54–72.5 Gy). Fourteen patients (8%) received ≥ 70 Gy, and four patients (2%) received < 60 Gy (range, 54–58.6 Gy).

In our dosimetric analysis comparing target volume and extent of peritumoral edema, we found 22 patients (37%) in the small-field group actually received radiotherapy including the peritumoral edema in the CTV unintentionally due to the initially narrow range of edema. Thus, the entire study cohort was divided into two groups, edema (+) (n = 130) and edema (−) (n = 37), according to whether or not the entire peritumoral edema was included within the CTV. Patient characteristics between the edema (+) and edema (−) group were compared (Table 1).

### Follow-up and outcome analysis

Survival rates and failure pattern according to the radiotherapy target volume were analyzed. All patients were followed-up until death or time of analysis. The median follow-up duration was 20 months (range, 2–99 months). Follow-up MRI was performed 4 weeks after the end of concurrent chemoradiotherapy, 23 days after the end of the 3rd and 6th TMZ cycles, every 6 months for the first 2 years after the end of Stupp’s protocol, and annually thereafter. Disease progression was diagnosed by radiologic findings as well as neurologic and clinical findings using the Response Assessment in Neuro-Oncology (RANO) criteria.

**Figure 1:**
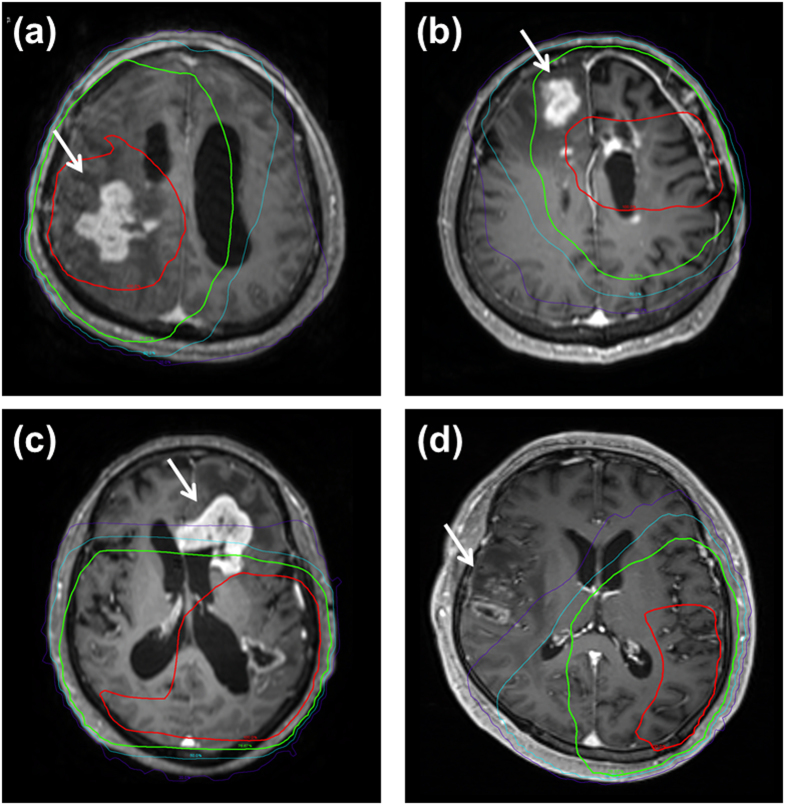
Examples of (**a**) infield GTV, (**b**) infield CTV, (**c**) marginal, and (**d**) outfield failures. Red and green lines represent isodose lines of 60-Gy and 46-Gy irradiation, respectively. Sky blue line represent an isodose line of 30-Gy and purple line represent an isodose line of 12 Gy.

To determine the location of treatment failures, simulation computed tomography (CT) images containing isodose volumes from radiotherapy planning were co-registered with the MRI that detected treatment failure using MIM software version 6.0.6 (MIM Software Inc., Cleveland, OH)^21^. Disease progression was categorized as followings according to location of T1 Gd-enhancing recurrent tumor: infield GTV (within the 60 Gy volume), infield CTV (outside the 60 Gy volume but within the 46 Gy volume), marginal failure (within 2 cm from the 46 Gy volume), outfield failure (all outside the CTV), and cerebrospinal fluid (CSF) seeding. Fig. 1 illustrates examples with isodose curves from the radiation treatment plan overlaid onto the MRI at the time of failure.

Patients with early enhancement within 3–5 months from the initiation of adjuvant therapies with a self-limited course and eventual resolution both clinically and radiographically were regarded as pseudoprogression^22^. Radiation necrosis (RN) was confirmed by operation or serial MRI images that showed improvement of a newly developed contrast enhanced lesion.

### Statistical analysis

Overall survival (OS) was estimated from the date of surgery to the date of death for any cause or of the last follow-up. Progression-free survival (PFS) was measured from the date of surgery to the date of recurrence or progression or to the last follow-up date for patients who did not experience these events. Survival differences were estimated using the log-rank test. Chi-squared analysis was performed to determine differences in patterns of failure between subgroups. SPSS version 20.0 (SPSS Inc., Chicago, IL, USA) was used and p-values < 0.05 were considered statistically significant.

## Results

### Survival and prognostic factor analysis

Disease progression was noted in 118 patients (71%) at a median 11 months (range, 1–68 months) from the date of surgery. A total of 133 deaths occurred among all patients, with 34 patients remaining alive until last follow-up (OS: median 36 months (range, 24–99 months). Twenty-six patients without disease progression on last follow-up images died later after aggravation of existing neurologic symptoms or pneumonia. In all patients, the median OS was 20 months, and 2- and 3-year OS rates were 43 and 27%, respectively. The median PFS was 15 months, and 2- and 3-year PFS rates were 35 and 23%, respectively.

In multivariate analysis, age (<60), EOR (GTR vs. non-GTR), and MGMT-methylation were independent prognostic factors for OS (p = 0.032, 0.004, 0.010, respectively), and EOR and MGMT-methylation were independent prognostic factors for PFS (p = 0.002, 0.012, respectively) (Table 2).

### Patterns of failure

**Figure 2:**
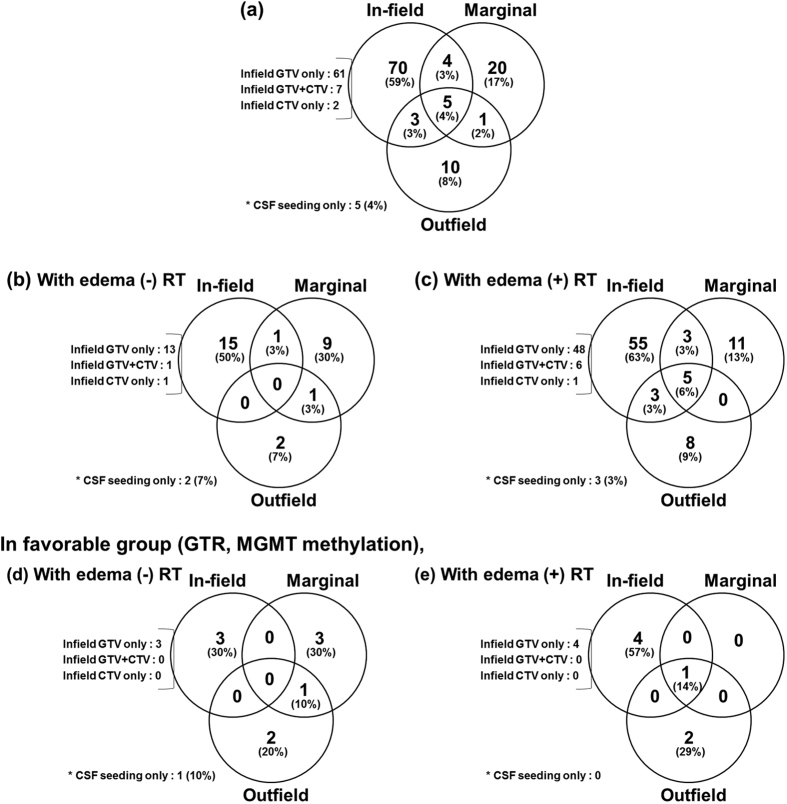
Patterns of failures (**a**) in all patients (n = 167), (**b**) in the edema (−) group (n = 37), and (**c**) in the edema (+) group (n = 130); Patterns of failures among patients with favorable prognosis (GTR, MGMT-methylation) (**d**) with edema (−) RT (n = 14) or (**e**) with edema (+) RT (n = 19).

Figure 2(a) illustrates the patterns of failure observed in all patient cohorts. The most common pattern was infield failure (82 patients, 69% of all failures). Almost all infield failures occurred within the GTV. GTV-only failures were noted in 61 patients (52%), GTV and CTV failures in seven (6%), and CTV-only failures in two (2%). Marginal failures were observed in 30 patients (26%) and outfield failures in 19 (16%). CSF seeding was identified in 14 patients (12%). Five patients were diagnosed with failures as CSF seeding only. The overall median times to infield, marginal, and outfield failure were 9 months (range, 1–68), 13 months (range, 2–56), and 14 months (range, 5–28), respectively.

### Relationship between radiotherapy volume and failure pattern

In the edema (−) group (n = 37), failures were identified in 30 patients (81%) (infield 53% > marginal 37% > outfield 10%). In the edema (+) group (n = 130), failures were identified in 88 patients (68%), and the rate of infield failures was higher and the rate of marginal failures was lower than the edema (−) group (infield 76% > marginal 22% > outfield 18%) (Table 3, Fig. 2(b),(c)). In chi-squared analysis, the marginal failure rate was significantly greater in the edema (−) group than the edema (+) group (37% vs. 22%, p = 0.050). The significance of peritumoral edema inclusion on marginal failure was maintained on multivariate analysis (odds ratio [OR], 0.277 [95% CI, 0.108–0.709]; p = 0.007) after adjusting for all potential confounding factors including radiation dose > 70 Gy, GTR, and MGMT methylation using logistic regression analysis. No significant difference was observed between the edema (−) and edema (+) groups in terms of infield GTV (47% vs. 61%, p = 0.288), infield CTV (7% vs. 8%, p = 0.138), and outfield-failures (10% vs. 18%, p = 0.572).

**Figure 3:**
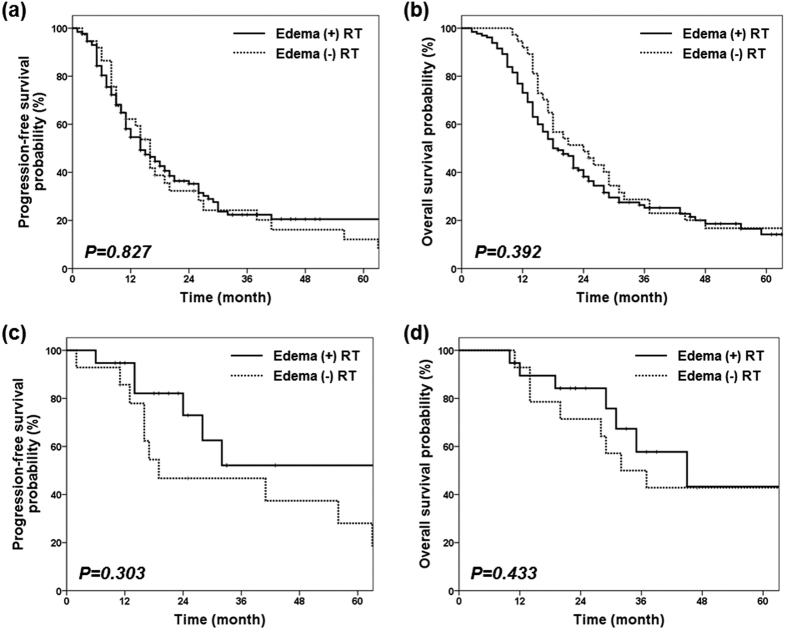
PFS and OS among all patients ((**a**) and (**b**)) and PFS and OS in patients with favorable prognosis (GTR, MGMT-methylation) ((**c**) and (**d**)) are compared according to inclusion of peritumoral edema in the radiotherapy target volume.

There was no significant difference in PFS and OS or timing of failure between the two subgroups. The two-year PFS and OS rates were 37 and 41% for the edema (+) group and 29 and 49% for the edema (−) group (p = 0.827, 0.392, respectively) (Fig. 3(a),(b)).

### Relationship between prognostic factors and failure pattern

Failure patterns were analyzed among subgroups of patients with favorable prognostic factors. In the GTR subgroup (n = 78), failures were identified in 50 patients (64%) (infield 68% > outfield 28% > marginal 24%). In the GTR + MGMT-methylation subgroup (n = 33), failures were identified in 17 patients (52%) (infield 47% > outfield 36% > marginal 29%). Although infield failure was the dominant pattern of failure in most subgroups, it was demonstrated that both marginal and outfield failures tended to increase and infield failures tended to decrease with the addition of a favorable prognostic factor (Table 3).

### Failure patterns according to the radiotherapy volume in the favorable prognostic group

Among 78 patients with GTR, total failure rate was decreased (58% vs. 81%, p = 0.060) and less marginal failures were noted (11% vs. 29%, p = 0.075) in the edema (+) group. Among 33 patients with GTR and MGMT-methylation, failure rate was further decreased (71% vs. 37% for edema (−) vs. (+) group, p = 0.049). The benefit of treating peritumoral edema on reducing marginal failure was more apparent (40% vs. 14% for edema (−) vs. (+) groups, p = 0.138) in this highly favorable prognostic group in comparison with that in the whole patient cohort. In particular, there was no marginal failure without infield failure when peritumoral edema was treated in this favorable group (Table 3, Fig. 2(d),(e)). However, there was no significant difference in survival outcome. The median PFS rates were 68 months in the edema (+) group and 19 months in the edema (−) group. The 2-year PFS rates were 82% for the edema (+) group and 47% for the edema (−) group (p = 0.303). The median OS rates were 45 months in the edema (+) group and 32 months in the edema (−) group. The 2-year OS rates were 84% for the edema (+) group and 71% for the edema (−) group (p = 0.433) (Fig. 3(c),(d)).

### Toxicity

Among 167 patients, pseudoprogression/RNs occurred in 66 patients (40%). Among patients with failure (n = 118), pseudoprogression/RNs occurred in 53 patients (45%). Treatment of peritumoral edema did not significantly increase the incidence of pseudoprogression/RN (edema (‒) vs. (+): 49% vs. 37%, p = 0.253). Radiation dose > 60 Gy was the only independent factor on multivariate analysis (OR, 2.7 [95% CI, 1.250–5.840]; p = 0.011).

## Discussion

Regarding the optimal radiotherapy volume, several retrospective studies suggested that a limited margin did not alter the failure patterns. In M.D Anderson Cancer Center, 48 patients were treated with a small field not intentionally including peritumoral edema. Recurrence patterns were compared between treatment planning parameters and newly created theoretical radiotherapy plans to include peritumoral edemas. Patterns of failure were identical between the two sets of plans with dominant central recurrence. It should be noted, however, only one-third (16/48) underwent GTR and 17 patients (35%) received adjuvant chemotherapy in this study^23^. A study from Italy also showed similar results supporting small field; however, while central recurrence was dominant (79/105, 75%) in 105 patients who received a current standard chemoradiation regimen with a small field, they observed higher rates of outfield failures in patients with MGMT methylation^14^. One UK study^24^ analyzed patterns of failure after TMZ-based chemoradiation with a small radiation field, and central relapses within the 2 cm remained predominant (77%). They concluded that a 2 cm margin from the primary enhancing mass is an appropriate technique. These results contrast with those of Gaspar *et al*.^25^, who found that smaller boost fields increased marginal tumor recurrences.

These pattern-of-failure studies, however, included patients who were treated with radiotherapy alone, radiotherapy with non-TMZ chemotherapy, and uncompleted TMZ chemotherapy, and only 9–30% of the patients received total tumor resection. Furthermore, no study included molecular data such as MGMT methylation status and stratified patients accordingly. Recent multicenter trials (RTOG 0525^26^ and CENTRIC EORTC 26071-22072)^27^ permitted both RTOG and EORTC protocols and showed no significant benefit of treating peritumoral edema in PFS and OS. However, detailed reports on the pattern of failure are lacking. Brandes *et al*.^15^ were the first to evaluate patterns of recurrences in the TMZ-based chemoradiation era. They observed that the failure pattern had shifted from local/marginal to distant recurrences outside the radiotherapy field, occurring in approximately 20% of cases. Furthermore, distant recurrences occurred significantly later than local recurrences (14.9 vs. 9.2 months, p = 0.02). Milano *et al*.^28^ also reported that the risk of central recurrence persists over time, but marginal, distant, and new infield recurrences tend to occur later in patients with longer survival times.

Regarding molecular prognostic marker status, 68–91% of patients without MGMT promoter methylation had local recurrence and 5.4–32% had distant recurrence, while fewer patients (58–65%) with MGMT promoter methylation had local recurrence, but more (31–42%) had distant recurrence^14^,^15^,^16^,^17^. The extent of surgical resection is one of the most important prognostic factors and could affect recurrence patterns. In the era of chemoradiation with TMZ, the local control rate can be improved with total tumor resection, and outfield recurrences can occur more frequently than before due to prolonged survival with local control. When small-field radiotherapy was applied to patients with GTR or STR, outfield failure increased up to 21.5%^15^. On the other hand, the patients with residual tumor after surgery showed significantly higher rates of local recurrence and lower rates of distant recurrence in comparison with the patients whose tumors were totally removed^24^,^29^,^30^. Although the characteristics of the patients were different in reported series and the definition of recurrence is not univocal, it has been suggested that favorable factors, including MGMT promoter methylation and GTR, prolonged PFS with decreased local recurrences and relatively increased distant recurrences.

All of our study patients received standard TMZ-based chemoradiotherapy, and a large portion of patients received extensive surgical resection (GTR 47%, STR 29%). Furthermore, MGMT methylation status was examined in most patients (87%). We also examined radiotherapy target volumes in all patients and determined whether or not actual inclusion of peritumoral edema influenced the recurrence pattern. Even though infield failure was the dominant pattern of failure in our study, the rates of marginal or outfield failure tended to increase in the patients with favorable prognostic factors (GTR and MGMT-methylation). We also suggest that inclusion of peritumoral edema in the radiotherapy target volume may reduce marginal failures without increasing radiation necrosis, especially in patients with totally resected and/or methylated MGMT.

Several limitations exist in our study. The relatively small number of marginal failures makes it difficult to find statistical significance when patients are subdivided according to prognostic factors and radiotherapy target volumes. Furthermore, the retrospective nature of our study makes it difficult to compare neurocognitive outcomes in relation to treatment volumes. A multi-institutional prospective study will be valuable in overcoming these limitations.

In conclusion, our study suggests that inclusion of peritumoral edema in the radiotherapy target volume may further reduce marginal failures without increasing toxicity, especially when favorable prognosis is expected by GTR and/or MGMT methylation. A prospective study with larger number of patients is needed to confirm our observations.

## Additional Information

**How to cite this article**: Choi, S. H. *et al*. Impact of Including Peritumoral Edema in Radiotherapy Target Volume on Patterns of Failure in Glioblastoma following Temozolomide-based Chemoradiotherapy. *Sci. Rep.* **7**, 42148; doi: 10.1038/srep42148 (2017).

**Publisher's note:** Springer Nature remains neutral with regard to jurisdictional claims in published maps and institutional affiliations.

## Figures and Tables

**Table 1:** Patient characteristics.

Characteristics	All patients (N = 167)	Edema (+) RT (N = 130)	Edema (−) RT (N = 37)	*p* value*
	No. (%)	No. (%)	No. (%)	
Age	Median 59 (range, 19–79)			
<60-year	82 (52)	66 (51)	21 (57)	0.52
≥60-year	80 (48)	64 (49)	16 (43)	
Sex				
M	85 (51)	65 (50)	20 (54)	0.66
F	82 (49)	65 (50)	17 (46)	
KPS	Median 80 (range, 30–100)			
<80	95 (57)	56 (43)	16 (43)	0.99
≥80	72 (43)	74 (57)	21 (57)	
Multiplicity				
Yes	28 (17)	24 (19)	4 (11)	0.26
No	138 (83)	106 (81)	33 (89)	
Gliomatosis				
Yes	12 (7)	10 (8)	2 (5)	0.64
No	155 (93)	120 (92)	35 (95)	
Ventricle involvement				
Yes	39 (23)	28 (22)	11 (30)	0.30
No	128 (77)	102 (79)	26 (70)	
MGMT methylation				
Methylation	59 (35)	43 (33)	16 (43)	0.40
Unmethylation	86 (52)	68 (52)	18 (49)	
Unknown	22 (13)	19 (15)	3 (8)	
Surgical extent				
GTR	78 (47)	57 (44)	21 (57)	0.70
STR	48 (29)	35 (27)	13 (35)	
PR	24 (14)	22 (17)	2 (5)	
Biopsy	17 (10)	16 (12)	1 (3)	
RT dose	Median 60 (range, 54–70)			
<70 Gy	153 (92)	126 (97)	27 (73)	<0.0001
≥70 Gy	14 (8)	4 (3)	10 (27)	

*Abbreviations*: KPS = Karnofsky performance status scale; MGMT = O6-methyl guanine-DNA methyltransferase; GTR = gross total resection; STR = subtotal resection; PR = partial resection; MRI = magnetic resonance imaging; RT = radiotherapy.

^*^p value in edema (+) RT group vs. edema (−) RT group.

**Table 2:** Univariate and multivariate analysis for patient survival.

Variables	OS			PFS		
	2-year (%)	Univariate	Multivariate	2-year (%)	Univariate	Multivariate
		*p* value	*p* value		*p* value	*p* value
Age		0.004	0.032		0.719	0.862
<60-year	50			35		
≥60-year	34			36		
Surgery		<0.001	0.004		<0.001	0.002
GTR	57			45		
<GTR	31			27		
RT dose		0.766			0.479	
<70 Gy	43			36		
≥70 Gy	48			29		
KPS		0.009	0.364		0.217	0.701
<80	33			33		
≥80	51			38		
Multiplicity		0.004	0.075		0.040	0.113
Yes	27			22		
No	47			38		
Ventricle involvement		0.021	0.363		0.103	0.507
Yes	33			23		
No	47			39		
MGMT methylation		0.002	0.010		0.005	0.012
Methylation	61			53		
Unmethylation	34			22		
Unknown	32			45		
Edema inclusion		0.535			0.657	
Yes	42			37		
No	49			29		
Pseudoprogression/RN		0.090			0.755	
Yes	58			38		
No	34			33		

*Abbreviations*: GTR = gross total resection; RT = radiotherapy; KPS = Karnofsky performance status scale; MGMT = O6-methylguanine-DNA methyltransferase; RN = radiation necrosis; OS = overall survival; PFS = progression-free survival; HR = hazard ratio; CI = confidence interval.

**Table 3:** Patterns of failure in each patient subgroup.

Pattern of failure	Failure No. (%)	Infield	Marginal	Outfield	CSF seeding only
		No. (%*)			
All patients (n = 167)	118 (71%)	82 (69%)	30 (26%)	19 (16%)	5 (4%)
Subgroups (according to prognostic factors)					
GTR (n = 78)	50 (64%)	34 (68%)	12 (24%)	13 (28%)	3 (6%)
MGMT methylation (n = 59)	37 (63%)	22 (59%)	10 (27%)	10 (27%)	4 (11%)
GTR + MGMT methylation (n = 33)	17 (52%)	8 (47%)	5 (29%)	6 (36%)	1 (6%)
GTR + MGMT methylation + Age < 60-year (n = 16)	7 (44%)	3 (43%)	2 (28%)	4 (57%)	0
Subgroups (according to RT target volume)					
Edema (+) RT (n = 130)	88 (68%)	66 (76%)	19 (22%)	16 (18%)	3 (3%)
Edema (−) RT (n = 37)	30 (81%)	16 (53%)	11 (37%)	3 (10%)	2 (7%)
Subgroups (according to prognostic factors & RT target volume)					
GTR & edema (+) RT (n = 57)	33 (58%)	26 (46%)	6 (11%)	10 (18%)	3 (9%)
GTR & edema (−) RT (n = 21)	17 (81%)	8 (38%)	6 (29%)	3 (14%)	4 (19%)
MGMT methylation & edema (+) RT (n = 43)	27 (63%)	19 (70%)	6 (22%)	7 (26%)	3 (11%)
MGMT methylation & edema (−) RT (n = 16)	10 (63%)	3 (30%)	4 (40%)	3 (30%)	1 (10%)
GTR + MGMT methylation & edema (+) RT (n = 19)	7 (37%)	5 (71%)	1 (14%)	3 (43%)	0
GTR + MGMT methylation & edema (−) RT (n = 14)	10 (71%)	3 (30%)	4 (40%)	3 (30%)	1 (10%)

*Abbreviations:* GTR = gross total resection; MGMT = O6-methyl-guanine-DNA methyltransferase; RT = radiotherapy; CSF = cerebrospinal fluid.

^*^% in all failures (Not the % in all patients).
